# Investigation of Ion Transport Parameters and Electrochemical Performance of Plasticized Biocompatible Chitosan-Based Proton Conducting Polymer Composite Electrolytes

**DOI:** 10.3390/membranes10110363

**Published:** 2020-11-21

**Authors:** Jihad M. Hadi, Shujahadeen B. Aziz, Salah R. Saeed, Mohamad A. Brza, Rebar T. Abdulwahid, Muhamad H. Hamsan, Ranjdar M. Abdullah, Mohd F. Z. Kadir, S. K. Muzakir

**Affiliations:** 1 Department of Medical Laboratory of Science, College of Health Sciences, University of Human Development, Kurdistan Regional Government, Sulaimani 46001, Iraq; jihad.chemist@gmail.com; 2 Hameed Majid Advanced Polymeric Materials Research Lab., Department of Physics, College of Science, University of Sulaimani, Qlyasan Street, Sulaimani 46001, Iraq; mohamad.brza@gmail.com (M.A.B.); rebar.abdulwahid@univsul.edu.iq (R.T.A.); ranjdar.abdullah@univsul.edu.iq (R.M.A.); 3 Department of Civil Engineering, College of Engineering, Komar University of Science and Technology, Sulaimani 46001, Iraq; 4 Charmo Research Center, Charmo University, Peshawa Street, Chamchamal, Sulaimani 46023, Iraq; salah.saeed@charmouniversity.org; 5 Department of Manufacturing and Materials Engineering, Faculty of Engineering, International Islamic University of Malaysia, Kuala Lumpur 53100, Malaysia; 6 Department of Physics, College of Education, Old Campus, University of Sulaimani, Kurdistan Regional Government, Sulaimani 46001, Iraq; 7 Centre for Foundation Studies in Science, University of Malaya, Kuala Lumpur 50603, Malaysia; hafizhamsan93@gmail.com (M.H.H.); mfzkadir@um.edu.my (M.F.Z.K.); 8 Material Technology Program, Faculty of Industrial Sciences & Technology, Universiti Malaysia Pahang, Lebuhraya Tun Razak, Gambang, Kuantan 43600, Malaysia; saifful@ump.edu.my

**Keywords:** solid polymer electrolyte, electrochemical impedance spectroscopy, ion transport parameters, TNM and LSV measurement, EDLC device

## Abstract

In this study, biopolymer composite electrolytes based on chitosan:ammonium iodide:Zn(II)-complex plasticized with glycerol were successfully prepared using the solution casting technique. Various electrical and electrochemical parameters of the biopolymer composite electrolytes’ films were evaluated prior to device application. The highest conducting plasticized membrane was found to have a conductivity of 1.17 × 10^−4^ S/cm. It is shown that the number density, mobility, and diffusion coefficient of cations and anions fractions are increased with the glycerol amount. Field emission scanning electron microscope and Fourier transform infrared spectroscopy techniques are used to study the morphology and structure of the films. The non-Debye type of relaxation process was confirmed from the peak appearance of the dielectric relaxation study. The obtained transference number of ions (cations and anions) and electrons for the highest conducting sample were identified to be 0.98 and 0.02, respectively. Linear sweep voltammetry shows that the electrochemical stability of the highest conducting plasticized system is 1.37 V. The cyclic voltammetry response displayed no redox reaction peaks over its entire potential range. It was discovered that the addition of Zn(II)-complex and glycerol plasticizer improved the electric double-layer capacitor device performances. Numerous crucial parameters of the electric double-layer capacitor device were obtained from the charge-discharge profile. The prepared electric double-layer capacitor device showed that the initial values of specific capacitance, equivalence series resistance, energy density, and power density are 36 F/g, 177 Ω, 4.1 Wh/kg, and 480 W/kg, respectively.

## 1. Introduction

Polymer electrolytes with H^+^ (or proton) as a charge carrier species were used in electrochemical energy storage device application [1,2]. Polymers offer a wide variety of applications, and their industry has grown more rapidly compared to other classes of materials. Among their applications, solid polymer electrolyte (SPE) has been extensively investigated. Essentially, it can be formed through dissolving inorganic salts in a polymer matrix comprising heteroatoms like O, N, S, and so on [3,4,5]. Chitosan (CS) has received a great deal of attention by means of a natural polymer due to its noteworthy advantages, including biodegradability, biocompatibility, safety, electrolytic characteristics, and high chemical resistance [6]. Also, CS backbone structure possesses multifunctional properties (i.e., OH and NH_2_), and the protonated amino group makes it capable of forming a high conductivity of the polymer electrolyte system [7]. The amine group (NH_2_) in the structure can act as an electron donor to interact with the inorganic salts. The nitrogen atom acts as a complexation site for the coordination of cations. The interaction between the inorganic salts with the CS leads to improvement of the amorphous structure [8]. The conductivity of ions is the dominant property of a polymer electrolyte and is improved by enhancing the amorphous nature. Because of these features, CS has the potential to be used in the application of energy storage devices like a host polymeric material, which in turn reduces environmental pollutions [9].

Polymer electrolytes offer a wide variety of applications in energy storage devices. The polymer electrolytes can be used for application in fuel cells. Polymer electrolyte fuel cell is of interest as a power source in vehicle and portable application owing to its eco-friendly and high energy efficiency [10]. Supercapacitors (SCs) are also a class of electrochemical energy storage devices considered as promising energy conversion devices for an extensive variety of applications. SCs are used in various applications, where the necessity is to store or release large quantities of energy in a little amount of time. Currently, the SCs are used mainly in electric vehicles, hybrid electric vehicles, and fuel cell vehicles like trains, passenger cars, and trolleybuses. Another field of SCs’ application are electronic devices such as volatile memory backups and uninterruptible power supplies. Another field of application are solar arrays or wind turbines, energy harvesting systems, where SCs have a supplementary role next to traditional batteries [11,12]. SCs are generally classified into pseudocapacitor (PC) and electrical double-layer capacitor (EDLC), which depends on their charge storage mechanisms [13,14]. EDLC consists of a couple of polarized porous electrodes and does not experience Faradaic reactions across the operating range of potential [15]. The EDLC function depends on the phenomenon involving forming an electrical double-layer at the interfaces between the electrolyte sample and electrodes [16]. In addition, through using the EDLCs, the device can be charged and discharged quickly within a few seconds, which is attributed to its storage mechanism [17]. 

Ali et al. [18] fabricated carbon nanospheres (NSs) using lablab purpureus seeds and they showed that the carbon NSs materials are useful in SCs electrode applications. The literature shows that various forms of carbons have been used as electrodes in fabricating of EDLCs owing to its excellent thermal and chemical stability with its abundance, including graphite, carbon nanotubes, carbon aerogel, and activated carbon [19,20,21]. On the other hand, the properties of the activated carbons (ACs) used as an active material greatly influence the performances of EDLCs, for instance, size and shape of pores, electrical conductivity, as well as surface functional groups [22,23]. However, the AC has been widely discovered for commercial EDLCs as a result of providing high values of capacitance due to its high microporosity [24]. 

Meanwhile, using the zinc metal complex (Zn(II)-complex) in the preparation of biodegradable polymer-based electrolytes contributes to improve the amorphous phase, which is promising for the ionic conduction mechanism. Asnawi et al. [25] showed that the addition of the Zn(II)-complex into the CS polymer electrolyte improved the EDLC device performance. The authors showed that the addition of Zn(II)-complex into the CS-based electrolyte improved an amorphous phase for ion conduction. Brza et al. [26,27] indicated that the inclusion of Ce(III)-complex into Poly(vinyl alcohol) (PVA) polymer electrolyte improved the amorphous phase and the performance of the EDLC device. The authors also indicated that the Ce(III)-complex improved the transport parameters of diffusion coefficient (*D*), charge carrier density (*n*), and mobility (*µ*) of cations and anions due to the enhancement of the amorphous phase [26,27]. 

Due to the presence of multi-hydroxyl groups (OH) in its structure, glycerol was used as an adequate plasticizer to improve conductivity. Low lattice energy, large size of anion, and small size of cation have decided the option of the metal salts. It has been established that ammonium salts possess good thermal stability and high ionic conductivity. Through doping with ammonium iodide (NH_4_I), and using glycerol plasticizer, the local viscosity of the polymer electrolyte matrix is reduced, thus promoting the mobility of ions (i.e., NH_4_^+^), which in turn increases the electrical conductivity [6,16,28]. In this paper, various electrical and electrochemical performances of CS-based polymer composite electrolytes (PCEs) have been investigated. The glycerol plasticizer further improved the amorphous phase and dissociates the extra ions to enhance the electrical conductivity. Thus, it is reasonable to use the relatively high conductance of the glycerolized CS:NH_4_I:Zn(II)-metal complex based-(PCEs) system as the electrode separator for fabricating the EDLC. 

## 2. Materials and Methods 

### 2.1. Materials 

In this study, CS with the average molecular weight of 35,000 g/mol has been used as a polymer material. Other raw materials, including ammonium iodide (NH_4_I) as a doping salt, acetic acid (CH_3_COOH) as a solvent, and glycerol (C_3_H_8_O_3_) as a plasticizer, were used to fabricate plasticized systems. All the chemicals were supplied by Sigma-Aldrich (Kuala Lumpur, Malaysia) without being purified. 

### 2.2. Electrolyte Preparation 

The preparation of the glycerolized CS:NH_4_I:Zn(II)-complex system involves the following procedure. At the beginning, 1 g of CS was dissolved into 50 mL acetic acid (1%) solution for around 3 h. Then, 40 wt.% fixed quantity of the ammonium iodide (NH_4_I) was added into the above mixture. The solution was stirred continuously at room temperature with a magnetic stirrer until a homogenous mixture was formed. After that, a fixed amount of 10 mL of Zn(II)-complex was added into the solution. The same procedure is used to fabricate Zn(II)-complex as indicated in our previous study, in the Materials and Methods Section in Reference [29]. Subsequently, glycerol with different concentration was added to the CS:NH_4_I:Zn(II)-complex and constantly stirred until a homogenous solution appeared. The glycerol concentrations were added and changed from 0 to 30 wt.%. The prepared samples were labeled as CSNZG0, CSNZG1, CSNZG2, and CSNZG3 for CS:NH_4_I:Zn(II)-complex filled with 0, 10, 20, and 30 wt.% of glycerol, respectively. Lastly, the solutions were poured into Petri dishes and covered with filter paper to avoid any contamination. The Petri dishes were left untouched to evaporate the solvent eventually so as to attain dry films. Table 1 summarizes the composition and designation of PCE samples. 

### 2.3. Electrochemical Impedance Spectroscopy (EIS) Analysis 

The data processing of impedance measurement was carried out at room temperature using a LCR meter (HIOKI 3531 Z Hi-tester, Nagano, Japan). The range of frequency was measured between (50 Hz ≤ *f* ≤ 5 MHz). The synthesized films were placed between two stainless-steel (SS) electrodes after the films were cut into circles with diameter of 2 cm under spring pressure. To measure the real (*Z*′) and imaginary (*Z*″) parts of complex impedance spectroscopy (Z*), the cell was connected to a computer software. 

### 2.4. Field Emission Scanning Electron Microscopy (FESEM) and Fourier Transform Infrared Spectroscopy (FTIR) Study

Field emission scanning electron microscopy (FESEM) was performed using a Hitachi SU8220 (Hitachi, Tokyo, Japan) at 500× magnification. The samples’ morphology was studied by the FESEM images. The films were analyzed using a Fourier Transform Infrared (FTIR) Spectrophotometer (Thermo Scientific, Nicolet iS10, Ashford, UK) that was fixed in the wavenumber range between 450 and 4000 cm^−1^ and having 2 cm^−1^ resolution.

### 2.5. Transfer Number Measurement (TNM) and Linear Sweep Voltammetry (LSV) Analysis 

The V & A instrument DP3003 digital direct current (DC) power supply has been employed to obtain the cation and anion transference number (*t_ion_*) and electronic transference number (*t_el_*) using the DC polarization method. The highest conducting composite electrolyte (i.e., CSNZG3) was kept between a pair of analogous stainless-steel (SS) blocking electrodes. A constant operating potential of 0.2 V was applied, and the cell was polarized at ambient temperature. To observe the highest operating voltage of the CSNZG3 system, the linear sweep voltammetry (LSV) has been done using a Digi-IVY DY2300 potentiostat. The LSV was measured at a scan rate of 10 mV/s in the voltage range from 0 to 2.5 V. The cell arrangement was the same as the TNM arrangements.

### 2.6. Fabricating EDLC

The creation of the EDLC electrodes comprises four consecutive stages. The first stage involves a dry mixing procedure, in which 3.25 g of activated carbon (AC) is mixed with 0.25 g of carbon black (CB) at 500 rpm for about 20 min. Additionally, 0.5 g of polyvinylidene fluoride (PVdF) is added to the 15 mL *N*-methyl pyrrolidone (NMP) solvent and mixed for around 60 min. The second stage was to dissolve AC-CB powder into a PVdF-NMP solution using a binder until a dark black solution appeared. The third stage is the coating of the produced black thick solution into an aluminum foil using a doctor blade. In the fourth stage, the coated aluminum foil was then dried in an oven (~60 °C) for several hours. Subsequently, the electrodes were located in a silica gel desiccator to eliminate extra moisture. Also, the electrodes were cut with an area of 2.01 cm^2^. The cell arrangement of the EDLC was performed by placing the highest conducting sample between two activated carbon electrodes. Then, the CR2022 coin cell was used to pack the electrolyte cell in a case of Teflon. To evaluate the energy storage mechanism, the preliminary test for the fabricated EDLC was performed at ambient temperature via cyclic voltammetry (CV). A Digi-IVY DY2300 potentiostat was employed at the different sweep rates in the potential range from 0 to 0.9 V. The EDLC charge-discharge profile was investigated using the Newer battery cycler at a current density of 0.5 mA/cm^2^.

## 3. Results and Discussion

### 3.1. Impedance Spectroscopy

**Figure 1:**
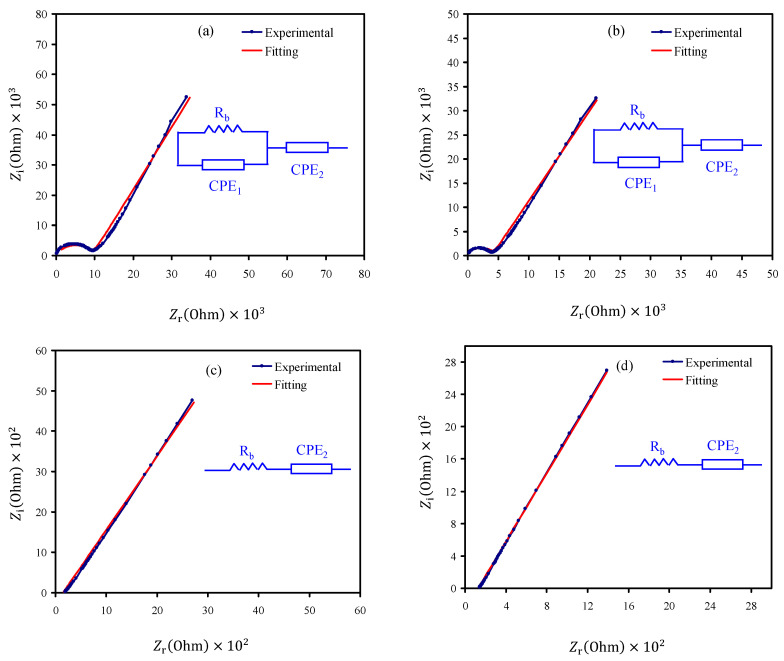
EIS plots for (**a**) CSNZG0, (**b**) CSNZG1, (**c**) CSNZG2, and (**d**) CSNZG3 electrolyte films.

Electrical impedance spectroscopy (EIS) is a familiar approach in electrochemistry for measuring both bulk transport behavior and electrochemical reactions on its superficial material [30]. It is a powerful technique to recognize the effect of different layers of polymers, chemicals, and composite electrodes. Its measurements will cover the key parameters of an electrochemical system, such as a change in current density, charge transfer resistance, as well as double-layer capacitances. Furthermore, the surface roughness and porosity of an electrode can also be evaluated using EIS [31]. The impedance spectra (*Z_i_* versus *Z_r_*) of the CSNZG0, CSNZG1, CSNZG2, and CSNZG3 composite electrolyte samples at room temperature are shown in Figure 1a–d. The normal finding for the EIS studies in polymer electrolytes usually involves two distinct regions: namely a spike and a semicircle at the region of low and high frequency, respectively. The high-frequency semicircle results from the conduction process in the bulk polymer composite electrolytes and it is represented by a parallel combination of bulk capacitance and bulk resistance. Whereas, the low-frequency spike characterizes the electrical double-layer capacitance at the blocking electrodes [32]. The charge accumulation at the region of the sample/electrode interfaces creates EDLC. One may notice that the impedance plots display a straight line at the low-frequency region which is due to electrode polarization (EP) [33,34]. One can obtain the bulk resistance of the electrolyte (*R_b_*) values due to the point from the straight line intercepts on the *Z_r_*-axis of impedance plots. Correspondingly, the following formula was used to calculate the ionic conductivity ($\sigma_{dc}$) of the composite polymer electrolytes [35]:

(1) $$\sigma_{dc}=\left(\frac{1}{R_{b}}\right)\times \left(\frac{t}{A}\right)$$

where *t* is the sample thickness, and *A* is the area of the electrolyte sample. It is noteworthy that the *R_b_* value was decreased with increasing of the glycerol concentrations and reached its minimum values at 30 wt.% of glycerol content (see Figure 1a–d) [36]. Earlier studies on different polymer electrolytes have shown that the DC conductivity was improved up to two orders using the glycerol plasticizer [4,16,21,37]. The DC ionic conductivity was determined and presented in Table 2. It was found that the electrolyte sample incorporated with 30 wt.% of glycerol displayed the highest ionic conductivity of 1.17 × 10^−4^ S/cm. Osman et al. [38] have attained the room temperature ionic conductivity of 4 × 10^−5^ S/cm using the CS-LiCF_3_SO_3_-ethylene carbonate (EC) system. Leones et al. [39] documented a result for the system of CS-glycerol incorporated with cyano-based ionic liquids. Ali et al. [40] fabricated porous nanocarbons (NCs) using bio-waste oil palm leaves. The porous carbon nanoparticles (NPs) showed superior supercapacitance (SPs) properties. Low resistance values were achieved in their study via fitting the impedance spectra representing the availability of these porous NCs as a precursor in the SCs electrode fabrication. Ali et al. [41] in another study fabricated porous carbon NPS with very high SPs value. Impedance spectra showed low resistance values of these materials, indicating their suitability for application in SCs electrodes.

The electrical equivalent circuit (EEC) model is used in the study of impedance spectroscopy as the EEC model is quick and provides an entire picture of the systems [26]. The EIS plots are interpreted in terms of the EEC including *R_b_* for the charge carriers in the samples and two constant phase elements (CPEs), which are CPE1 and CPE2, as shown in the inset of Figure 1a–d. The high-frequency region shows the *R_b_* and CPE1 connection in parallel, whereas the low-frequency region shows CPE2, i.e., the formed double-layer capacitance between the SPE and electrodes. The impedance of CPE (*Z_CPE_*) is expressed as [26]:

(2) $$Z_{CPE}=\frac{1}{C\omega^{p}}\left[cos\left(\frac{\pi p}{2}\right)-i\;sin\left(\frac{\pi p}{2}\right)\right]$$

In Equation (2), *C* is the CPE capacitance, *ω* is the angular frequency, and *P* relates to the deviation of the vertical axis of the impedance plots. The real part (*Z_r_*) and imaginary part (*Z_i_*) of complex impedance (Z*) related with the EEC (inset of Figure 1a,b) are expressed as:

(3) $$Z_{r}=\frac{{R_{b}}^{2}\left(Z1\right)+R_{b}}{2R_{b}\left(Z1\right)+{R_{b}}^{2}{C_{1}}^{2}\omega^{2p1}+1}+\frac{Z2}{C_{2}\omega^{p2}}$$

where, $Z1=C_{1}\omega^{p1}cos(\frac{\pi p_{1}}{2})$ and $Z2=cos\left(\frac{\pi p_{2}}{2}\right)$

(4) $$Z_{i}=\frac{{R_{b}}^{2}\left(Z3\right)}{2R_{b}\left(A1\right)+{R_{b}}^{2}{C_{1}}^{2}\omega^{2P1}+1}+\frac{Z4}{C_{2}\omega^{p2}}$$

where, $Z3=C_{1}\omega^{p1}sin\left(\frac{\pi p_{1}}{2}\right)$ and $Z4=sin\left(\frac{\pi p_{2}}{2}\right)$

All the circuit element parameters that are used for impedance plots’ fitting for all the samples are shown in Table 3. In the Cole-Cole plots, at higher glycerol concentration (Figure 1c,d), the semicircle disappeared, indicating that only the resistive component of the electrolyte systems exists [26]. The disappearance of the semicircle in the CSNZG2 and CSNZG3 systems is because most of the cations and anions in the bulk of the electrolyte move in opposite directions toward the electrodes to form the double layer. In such case, the *Z_r_* and *Z_i_* associated to the EEC are expressed as:

(5) $$Z_{r}=R+\frac{Z2}{C_{2}\omega^{p2}}$$

(6) $$Z_{i}=\frac{Z4}{C_{2}\omega^{p2}}$$

As the (CSNZG0 and CSNZG1) impedance data consists of a spike/tail and a semicircle, the transport parameters of diffusion coefficient (*D*), mobility (*μ*), and number density (*n*) of cations and anions are computed by the equations below [26]. 

The *D* of the cations and anions in CSNZG0 and CSNZG1 systems is computed by the relation,

(7) $$D=\left(\frac{{\left(K_{2}\varepsilon_{o}\varepsilon_{r}A\right)}^{2}}{\tau_{2}}\right)$$

where *ε_r_* is the dielectric constant, *ε_o_* is the permittivity in vacuum, and *τ_2_* is the reciprocal of angular frequency corresponding to the minimum in *Z_i_*. 

The mobility (*µ*) of the cations and anions is calculated via the relation below,

(8) $$\mu =\left(\frac{eD}{K_{b}T}\right)$$

where *T* is the absolute temperature and *k_b_* is the Boltzmann constant. 

Since conductivity (*σ_DC_*) is shown by

(9) $$\sigma_{Dc}=ne\mu ,$$

so, the number density of the cations and anions (*n*) is computed using Equation (10):

(10) $$n=\left(\frac{\sigma_{dc}K_{b}T\tau_{2}}{{\left(eK_{2}\varepsilon_{o}\varepsilon_{r}A\right)}^{2}}\right)$$

As the impedance data of CSNZG2 and CSNZG3 consists of a spike, the *D* is calculated by Equation (11) [26]:

(11) $$D=D_{{}^{\circ}}exp\{-0.0297{\left[lnD_{{}^{\circ}}\right]}^{2}-1.4348lnD_{{}^{\circ}}-14.504\}$$

where

(12) $$D_{{}^{\circ}}=\left(\frac{4k^{2}l^{2}}{{R_{b}}^{4}\omega 3_{min}}\right)$$

where l is the electrolyte thickness, and *ω_min_* is the angular frequency corresponding to the minimum *Z_i_*. Table 4 shows the cation and anion transport parameters and the *ω_min_* values for the electrolyte system. 

Based on Table 4, the value of *D* is observed to increase as the glycerol concentration increases from 10 to 30 wt.%. A similar trend is shown by *μ* as indicated in Table 4 where μ increases. The increase of *μ* and *D* is ascribed to the enhancement of chain flexibility with the glycerol existence [26]. When the glycerol concentration is increased, the values of *D*, *μ*, and *n* are increased, which causes an increase in conductivity. This is because the more glycerol addition dissociates more salts to free cations and anions, thus increasing the number density of cations and anions [26].

**Figure 2:**
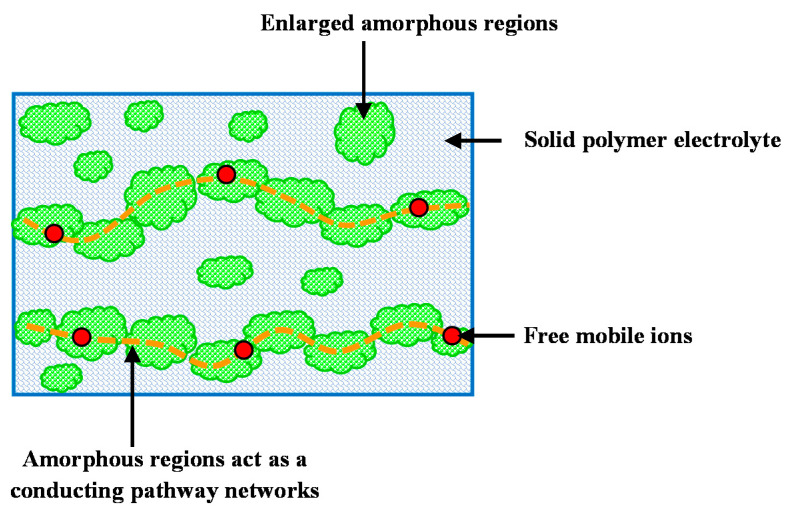
Percolative network of disordered regions produces rapid ion transport pathways for the mobile ions [5].

The amorphous nature of polymer electrolyte is enhanced by adding salts into the polymer. The ionic conductivity and enhancement of amorphousity is associated to the greater mobility of ions and ions’ diffusivity in the amorphous nature owing to the limited energy barrier. It was indicated in the earlier study [42] that the enhancement of the amorphous phase is valuable in local chain segmental movements, which will promote the movement of ions and thus, improve the conductivity of ions. It was also indicated in another study [43] that when the polymer film amorphous structure improves, there is an improvement in mobility of ions as larger free volume is provided in the polymer structure. This brings about an improvement in polymer chains’ segmental motions owing to the increase in the flexibility of the polymer chains. Thus, there is an increase in the polymer electrolyte conductivity. The increase of DC conductivity with glycerol amount is rationalized by the presence of a percolating system for high plasticizer contents [5]. Figure 2 indicates the effect of plasticizer on the percolative behavior of the transport of ions.

### 3.2. Field Emission Scanning Electron Microscopy (FESEM) and Fourier Transform Infrared Spectroscopy (FTIR)

**Figure 3:**
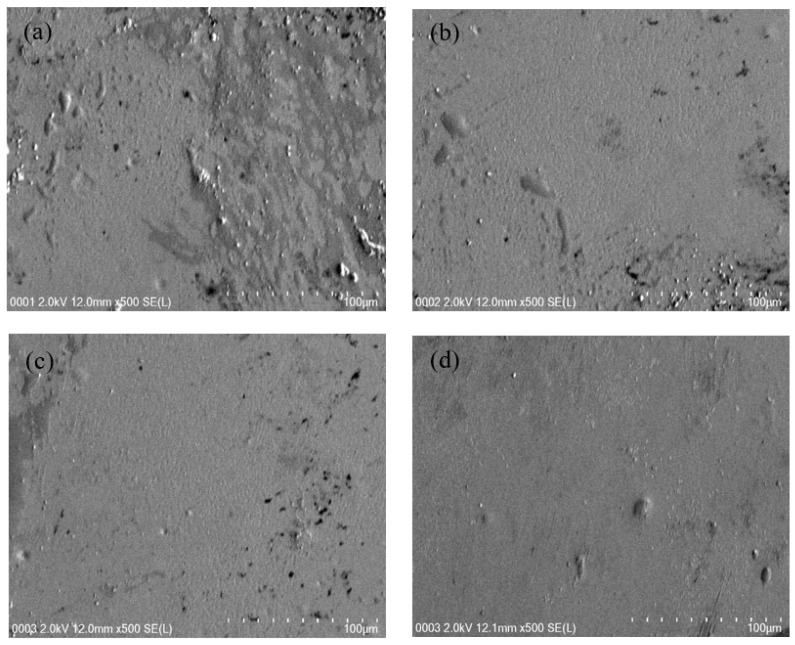
Field emission scanning electron microscopy (FESEM) images (**a**) CSNZG0, (**b**) CSNZG1, (**c**) CSNZG2, and (**d**) CSNZG3 electrolyte films.

Field emission scanning electron microscopy (FESEM) images were taken at 500× magnification for the films to support the EIS results. The FESEM for the films are indicated in Figure 3a–d. When there is incorporation of 10 and 20 wt.% glycerol into the electrolyte system, salts were protruded within the samples’ surface, as indicated in Figure 3a,b. It is evident from the FESEM images that the protrude salts in the CSNZG3 system are not clearly observed as the concentration of glycerol increased to 30 wt.% in comparison with the other systems. The highest plasticized electrolyte system exhibits smooth and uniform surface morphology without any phase separation. The FESEM images are in good agreement with the EIS results. It is documented that the smooth morphology appearance is related to the improvement of the amorphous phase of the electrolyte system [44]. The smooth surface electrolytes will assist conducting ions to transfer easily, and therefore decrease the bulk resistance and increase the conductivity of ions [44].

**Figure 4:**
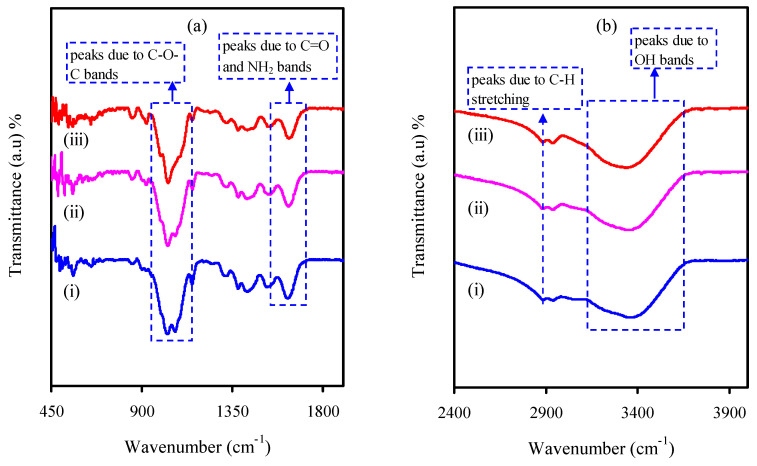
Spectra of (i) CSNZG1, (ii) CSNZG2, and (iii) CSNZG3 electrolyte films in the range (**a**) 450 to 1900 cm^–1^ and (**b**) 2400 to 4000 cm^–1^.

The FTIR spectra for the plasticized electrolyte systems are shown in Figure 4a,b. From the outcomes achieved in this study, a strong peak was concentrated at about 2890 cm^−1^, which is associated to the CH stretching mode [45]. Though, it is seen that that the intensity of the peak is decreased with increasing glycerol amount. Another remark from the literature is that polymer chains comprising electronegative atoms (N or O) in the repeating units can serve as solvents for the salts [46]. This is indicated by the attractive reaction happening among the chains and the cations. Furthermore, it was indicated that chitosan is featured by a pair of hydroxyl groups (OH) and a single amino group (NH_2_) in each repeating units [47]. In a previous study [45], appearances of a wide peak at about 3359 cm^–1^ are ascribed to –OH stretching mode. The peak at about 1013–1060 cm^−1^ is related to C–O–C stretching modes [45]. As evidence, both peak shifting and change in intensity produce robust evidence about the complex creation among the dopant and the polymer. Each film is characterized by the key features of absorption peaks, such as change of O=C–NHR, NH_2_, and OH groups of CS [45]. From Figure 4, it is obvious that shifts happen in the direction of the lower wave numbers in the bands of O=C–NHR, NH_2_, and OH groups. This creates more understanding into the complex formation among the electrolyte components and the polymer.

### 3.3. Dielectric Properties

#### 3.3.1. Study of Dielectric Constant and Dielectric Loss

To investigate the conductivity behavior of polymer electrolytes, the study of dielectric analysis is important [48]. Its measurement plays a vital role in elucidating the molecular relaxation behavior associated with its ionic conductive features upon frequency [49]. Based on the plots of dielectric constant (*ε*′) and dielectric loss (*ε*″), the dielectric properties of the composite samples were determined using the equations in References [1,2].

**Figure 5:**
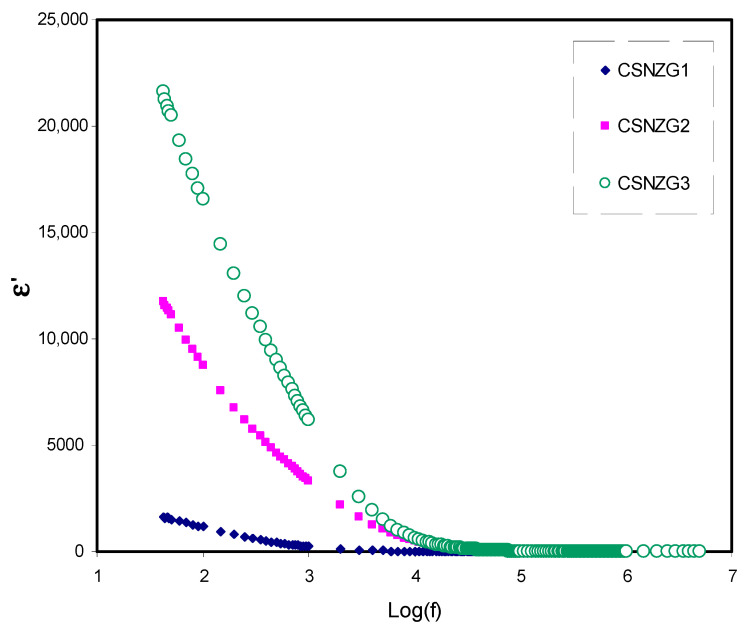
Variation of dielectric constant (*ε*′) versus frequency for the CSNZG1, CSNZG2, and CSNZG3 composite electrolyte samples at room temperature.

**Figure 6:**
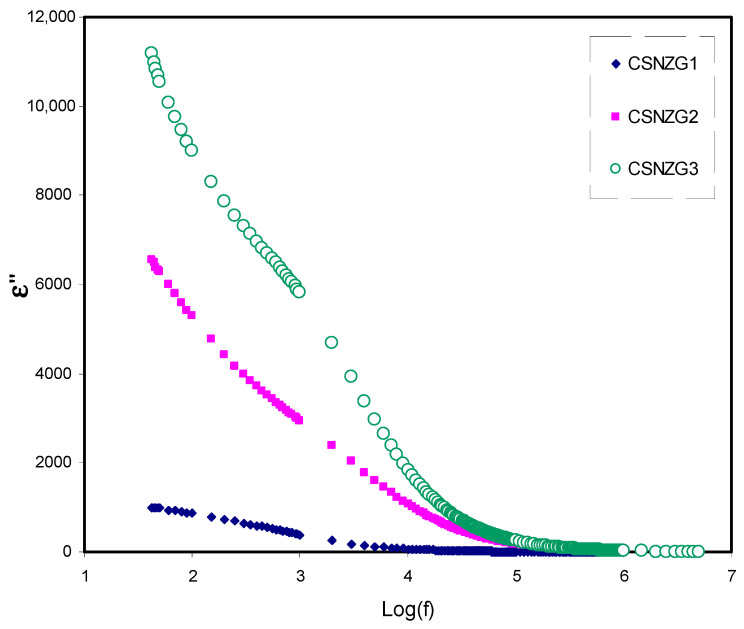
Variation of dielectric loss (*ε*″) versus frequency for the CSNZG1, CSNZG2, and CSNZG3 composite electrolyte samples at room temperature.

Figure 5 and Figure 6 show the frequency dependence of dielectric constant (*ε*′), and dielectric loss (*ε*″) at room temperature for the CSNZG1, CSNZG2, and CSNZG3 composite electrolyte films, respectively [50,51,52]. Both figures of dielectric constant and dielectric loss have the same behavior. The plots show the reduction in the dielectric constant and dielectric loss with increasing frequency, while at low-frequency, both dielectric parameters are quite high. This phenomenon indicates the occurrences of space charge effects and electrode polarization at the boundary between the electrodes and electrolyte. Therefore, the periodic reversal of the electric field at high frequency happens more rapidly, which reflects no dispersion of excess ions in the field direction. Consequently, polarization reduces due to charge aggregation, resulting in the observed decrease in dielectric permittivity [53,54,55,56]. It is interesting to conclude that further increase of the concentration of glycerol in the system leads to raising the values of ε′ and ε″ at lower frequency regions. Also, the CSNZG3 composite electrolyte film designates the higher ionic conductor due to its sharp rise in the ε′ value [57,58].

#### 3.3.2. Electric Modulus Study 

It is of a great opportunity to investigate relaxation processes of polymeric systems using dielectric spectroscopy. The complex dielectric function (ɛ*) comprises dielectric constant and loss, which are very sensitive for the applied frequency and structure of the materials [59,60,61]. At high frequency, both real (*M*′) and imaginary (*M*″) parts of complex modulus are relatively high, owing to the electrode polarization (EP) effect [62,63]. Macedo et al. have established a formalism of electric modulus to minimize the impact of EP [64]. Interestingly, the ion movement in solid polymer electrolytes can perturb the surrounding electric potential. This ion behavior under applied electric potential condition lead to non-exponential decay and distribution of relaxation time [65]. The reciprocal of the permittivity in the complex form is equal to complex electric modulus (M*) [66], and can be determined using the equations in References [1,2].

**Figure 7:**
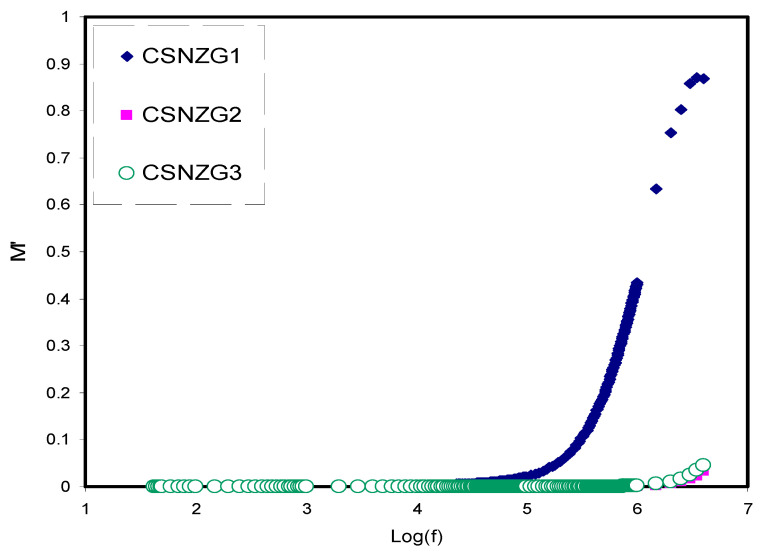
Variation *M*′ along with frequency for the CSNZG1, CSNZG2, and CSNZG3 composite electrolyte samples at ambient temperature.

**Figure 8:**
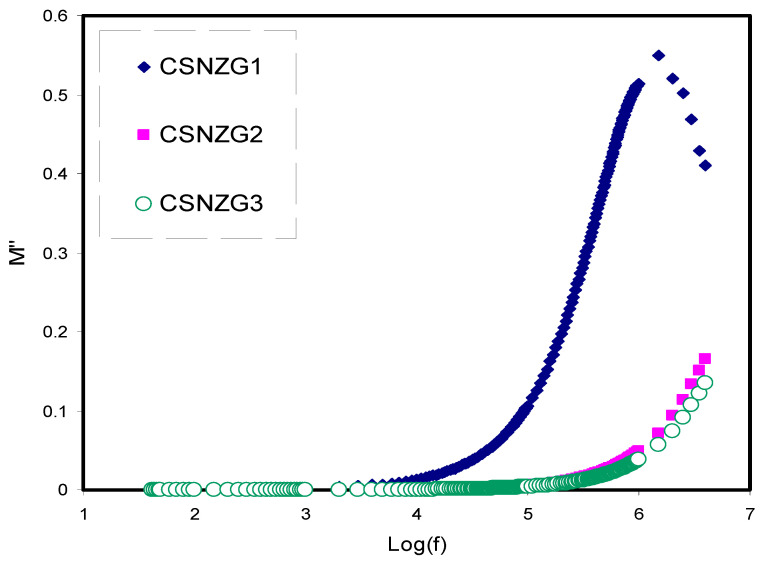
Variation of *M*″ along with frequency for the CSNZG1, CSNZG2, and CSNZG3 composite electrolyte samples at ambient temperature.

Figure 7 and Figure 8 reveal the frequency dependence of *M*′ and *M*″ of all the composite electrolyte films. At the low-frequency region, both *M*′ and *M*″ are small, indicating the absence of electrode polarization contribution in the electric modulus study [67,68]. It is interesting to notice that *M*′ reaches to a maximum value at the high-frequency region. This is due to decreasing the dielectric constant to a minimum value at the high-frequency region, as shown in Figure 5, and thus *M*′ reaches its maximum value (*M_∞_* = 1/*ɛ_∞_*) [69]. Figure 8 shows the frequency dependence of *M*″ at different concentrations of plasticizer. At the high-frequency region in the imaginary part of modulus (*M*″) spectra, a distinct relaxation peak is observed as a result of conductivity processes, while no peak is seen in the dielectric loss spectra, as shown in Figure 6. This confirms the contribution of both ionic and polymer segment motions in the conduction process [70,71,72]. As a consequence, the conduction process in the polymer electrolytes involves charge transport via ion migration alongside segmental motion.

### 3.4. TNM Analysis

**Figure 9:**
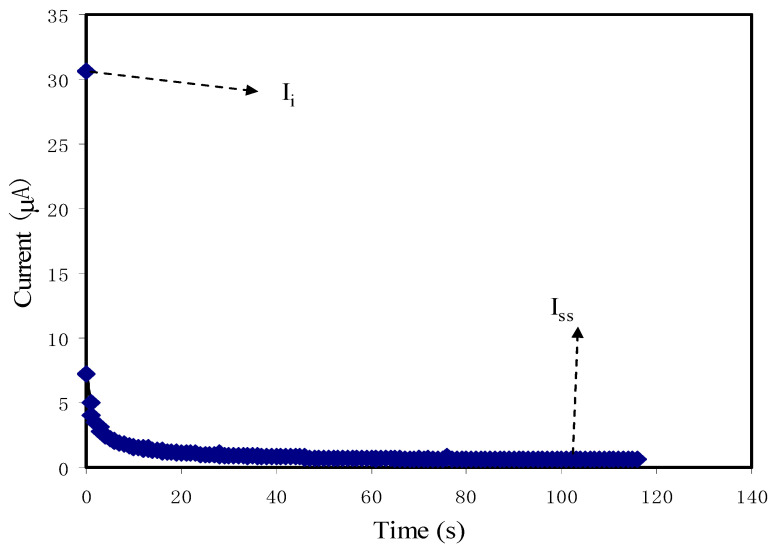
Current versus time for the CSNZG3 electrolyte sample.

In the composite SPEs, the determination of dominant cation and anion ions can be characterized based on the transference number measurement (TNM). The current was measured through the circuit until it reached a saturation state. The curve of polarization current versus time for the highest conducting system (i.e., CSNZG3) at the working voltage of 0.2 V is shown in Figure 9. It has been reported that the polymer electrolyte system is governed by cations and anions when the ionic TNM is close to the ideal value of unity [73,74]. In the fabrication of EDLC, it is crucial to confirm which species is the main charge carrier to the overall value of conductivity. The following equations are used to calculate the transference number of cations and anions (*t_ion_*) and electrons (*t_el_*):

(13) $$t_{ion}=\frac{I_{i}-I_{ss}}{I_{i}}$$

(14) $$t_{el}=1-t_{ion}$$

where *t_ion_* is the transference number of cations and anions, *t_el_* is the electron transference number, *I_i_* is the initial current holds electrons and cations and anions, and *I_ss_* stands for the steady-state current due to electrons only [75,76]. From the graph, an extreme drop in the initial current (31 µA) is observed and reduced as a function of time. The large value of current at the beginning is because the electrons, cations, and anions are involved. Cell polarization happens when it reaches the steady state, whereas transfer of the rest of the current is only due to electrons. This is because the stainless-steel electrodes make a barrier for the cations and anions, however they permit the electrons to move through [26]. The current decrement versus time is mainly due to the reduction of cation-carrier and anion-carrier as well as increase the electronic movement in the composite electrolyte system [77]. As a consequence, in the completely depleted charge carriers, the cell becomes constant. Based on the Equations (13) and (14), the measured *t_ion_* and *t_el_* for the sample incorporated with 30 wt.% glycerol were found to be 0.98 and 0.02, respectively [78]. The result reveals that the highest conducting sample of the glycerolized CS:NH_4_I:Zn-metal complex system is predominantly due to ions [79]. The TNM finding in this study is similar to the system of chitosan-NH_4_Br-glycerol as documented by Shukur et al. [80].

### 3.5. LSV Analysis

**Figure 10:**
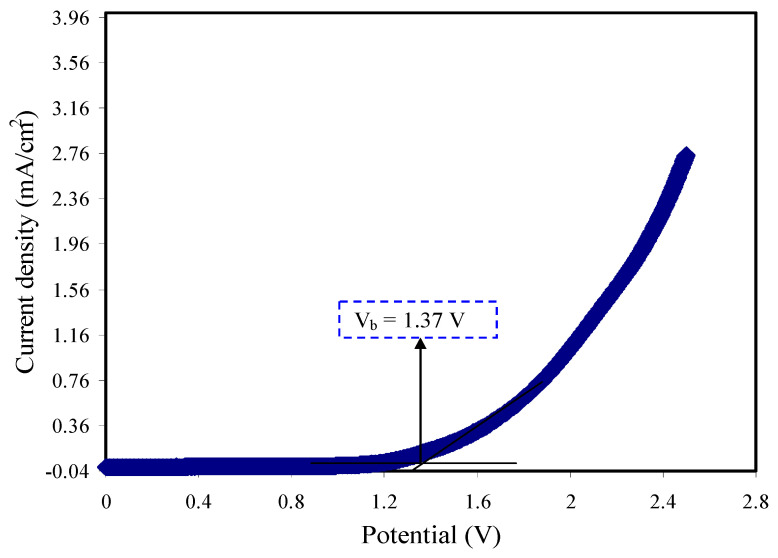
Linear sweep voltammogram (LSV) plot for the highest conducting CSNZG3 electrolyte sample.

The use of the linear sweep voltammetry (LSV) technique is to determine the electrochemical stability of the CSNZG3 sample. The EDLC of polymer electrolytes is supposed to be stable and display no peak degradation over the applied potential range [81]. Figure 10 represents the electrochemical stability window of the CSNZG3 electrolyte film at room temperature. A working voltage was measured in the range between 0 and 2.5 V at the scan rate of 10 mV/s. The LSV plot shows no obvious current in the range from 0 to 1.2 V. As the potential reached 1.3 V, the current was observed to rise steadily. This is indicating the sample decomposition at the surface of the inert electrode [82]. It was reported that the required value of the standard stability window of SPEs in the application of protonic devices is ~1 V [83]. Thus, the prepared CSNZG3 sample possesses adequate anode stability to be applied in electrochemical devices. The breakdown voltage for the starch-chitosan-NH_4_I-glycerol system was 1.9 V, as investigated by Yusof et al. [84]. In our previous study, a high breakdown voltage (V_b_) for the glycerolized CS:NH_4_F:Zn(II)-complex system was observed [25]. There may be some possible interpretation for the different values of V_b_ obtained in the present work compared to our reported one. It could be due to the ionic radius of F^−^ being smaller compared to I^−^, which makes it possible for better intercalation into the layered cathode structure. Thereby, a weak interaction between NH_4_^+^ with I^−^ is observed relative to NH_4_^+^ with F^−^ [85,86]. The lattice energy of NH_4_I is lower than NH_4_F [87], and thus systems impregnated with NH_4_I may possess lower V_b_. 

### 3.6. Cyclic Voltammetry (CV) Analysis

Over decades, diverse methods in electroanalytical chemistry have been developed. The extreme importance was achieved by cyclic voltammetry (CV) from both the physics and chemistry viewpoints. It is a strong and widespread electrochemical technique to characterize the oxidation-reduction process of molecular materials. Except for rigorously analytical issues, CV has already succeeded classical polarography. Its range of applications was extended from the study of basic redox procedures in organic and inorganic chemistry to the investigation of macromolecular chemistry and biochemistry of the multi-electron transfer process. However, molecular electrochemistry has become a key research technique aimed at improving technologies for clean energy sources [87,88]. The electrical capacitor performances and its charge storage will usually be evaluated with cyclic voltammetry (CV) at specific interfaces in the cathodic and anodic regions [89]. Hegde et al. [90] synthesized carbon NSs by a catalyst-free pyrolysis method from bio-waste sago bark. The capacitive behavior of the carbon NSs was proven by the CV profile at various scan rates which showed nearly rectangular-like shape. The author showed that the fabricated carbon NSs are suitable in SCs electrode application.

**Figure 11:**
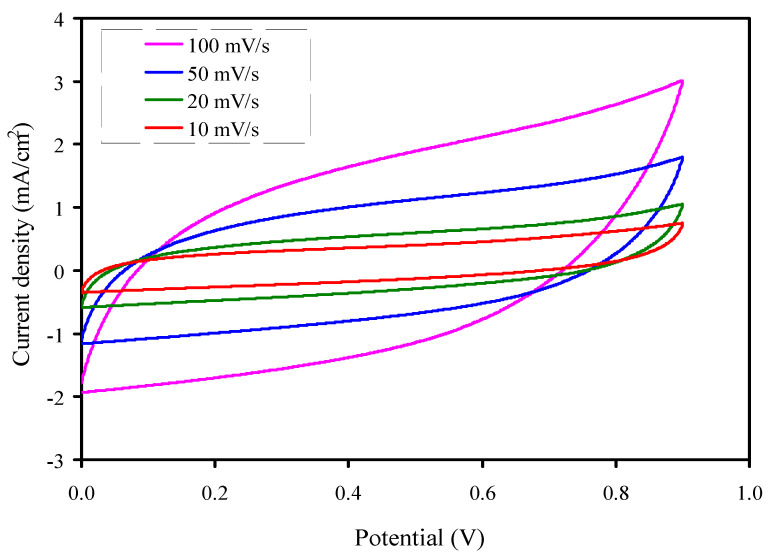
CV curve of the EDLC measured for the CSNZG3 sample at different sweep rates in the potential range of 0 to 0.9 V.

Figure 11 shows the CV responses for the highest conducting sample, where the potential range was measured from 0 to 0.9 V, at various scan rates of 10–100 mV/s. The CV profile of the constructed EDLC displays a leaf-like shape at the high scan rates, while its shape turned into an almost rectangular shape as the scan rate decreased [25,91]. The internal resistance and carbon porosity is the reason for the deviation from rectangular shape, thereby producing a current dependence of potential. The specific capacitance (*C_p_*) value from the CV profile can be determined using the following equation:

(15) $$C_{p}=\int_{V_{1}}^{V_{2}}\frac{I\left(V\right)dV}{2mS\left(V_{2}-V_{1}\right)}$$

where *I*(*V*)*dV* is the area of the CV plot, which was determined using the Origin 9.0 software via the function of integration. *V*_1_ and *V*_2_ are the initial voltage and final voltage, respectively. *m* stands for the mass of active material used, and *S* is the scan rate [92]. It is interesting to note that the CV plots possess no visible redox reaction peaks over its entire potential range. It is also obvious that the variation of scan rates influenced the shape of the CV plots and *C_p_* values (see Table 5). At higher scan rates, the amount of accumulated charges on the surface of the electrodes is decreased, due to the contribution of little charges for polarization, thus, the *C_p_* value is reduced [93,94,95]. At a lower scan rate, the rectangular-like shape of the fabricated EDLC is an indication for the fact that the characteristics of the EDLC are near to an ideal capacitor behavior. Arof et al. [96] achieved the *C_p_* value of 35 F/g for the polymer electrolyte system of CS/iota-carrageenan-based EDLC. The *C_p_* value of 32.69 F/g was obtained for the system of glycerolized PVA:CS:Mg(CF_3_SO_3_)_2_ at 10 mV/s [97].

### 3.7. Analysis of Charge-Discharge Characteristic

**Figure 12:**
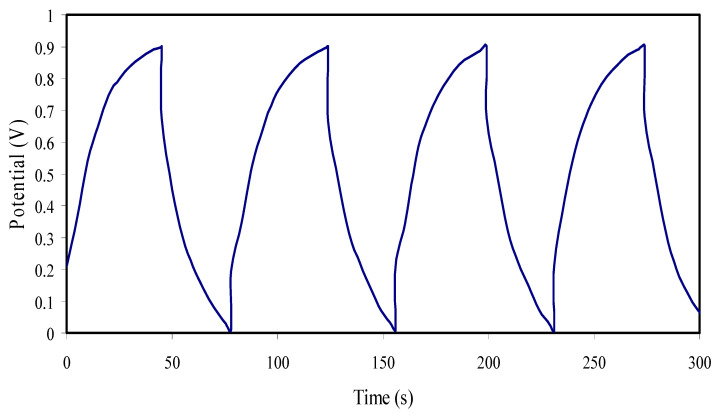
Galvanostatic charge-discharge performances of the constructed EDLC for the initial cycles.

A galvanostatic charge-discharge (GCD) profile of the fabricated EDLC device for the maximum conducting composite electrolyte containing 30 wt.% of glycerol is illustrated in Figure 12. The applied current density is 0.5 mA/cm^2^ over 400 cycles in the potential range between 0 and 0.9 V at room temperature [98]. The EDLC charge and discharge curve displays as almost linear, which indicates the capacitive behavior of the EDLC [99].

One may notice the rapid drop in the potential (V_d_) of the assembled EDLC before the initiation of the discharging process. It is obvious that a perfect triangle shape of the charge-discharge curve cannot be obtained. The deviation from the ideal triangle shape may be related to the influence of internal resistance, bulk electrolyte, and roughness of activated carbon [100,101].

**Figure 13:**
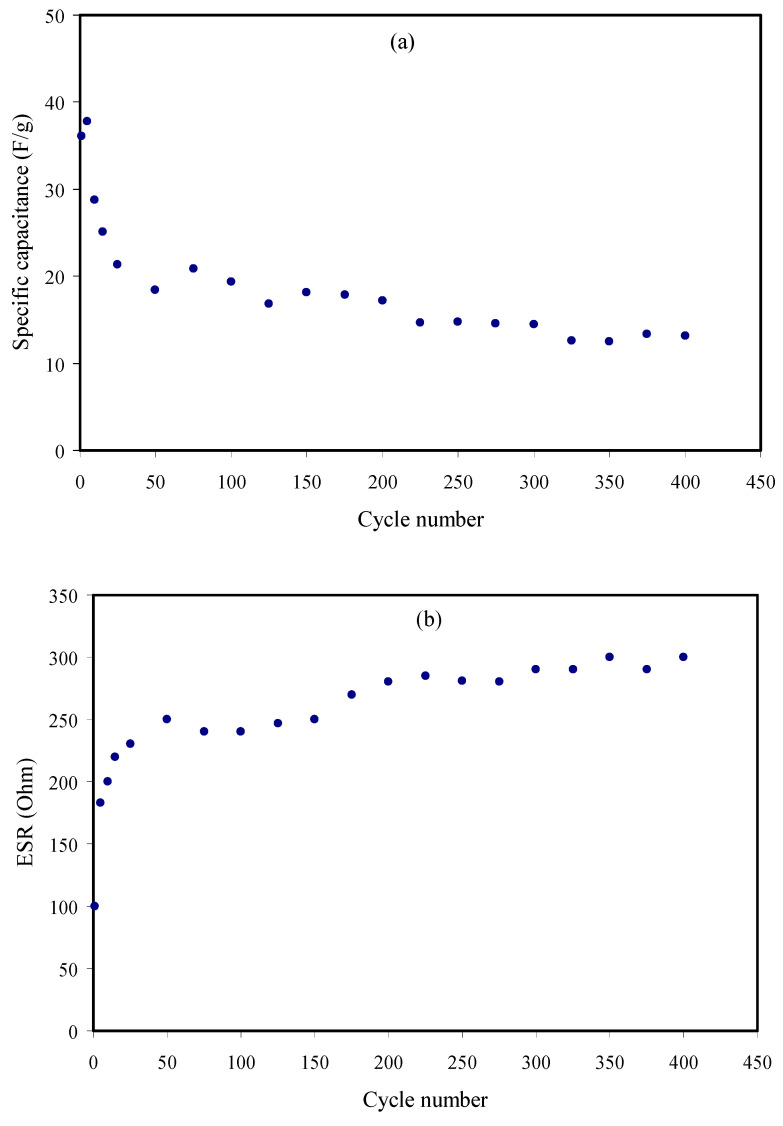

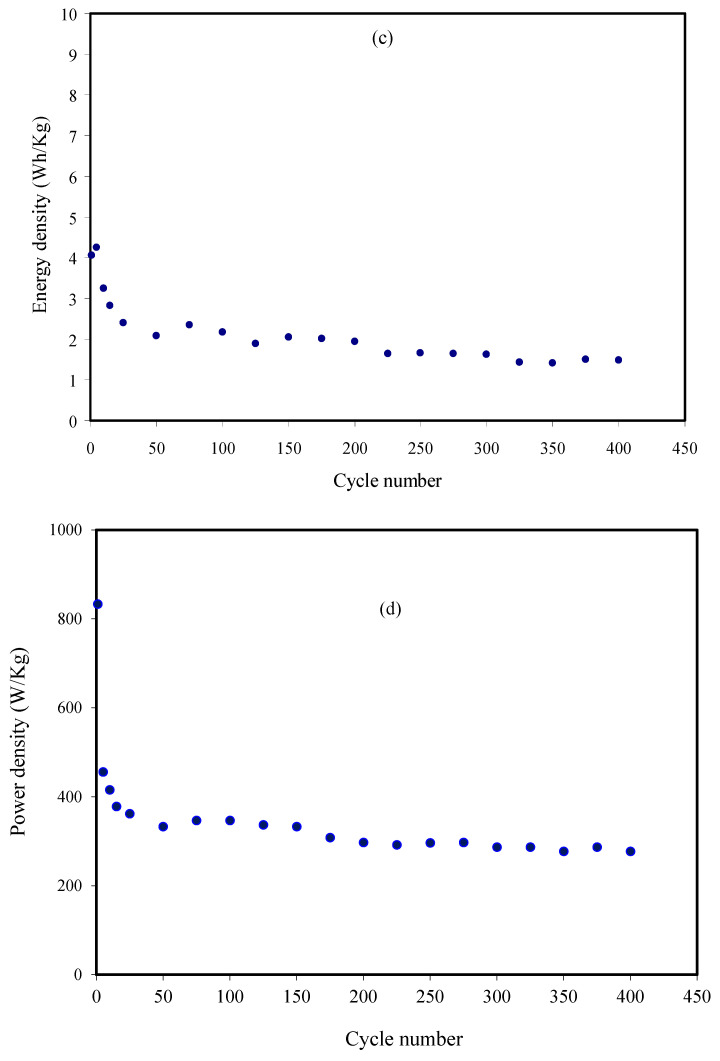
EDLC parameters of (**a**) specific capacitance (*C_s_*), (**b**) equivalence series resistance (*ESR*), (**c**) energy density (*E_d_*), and (**d**) power density (*P_d_*) versus cycle number.

The capacitance is the ratio between the changes of electrical charge to the change of the potential within a system. Figure 13a represents the variation of the discharge-specific capacitance (*C_s_*) of the constructed EDLC up to 400 cycles. The value of the *C_s_* of the EDLC was calculated from the following equation:

(16) $$C_{s}=\frac{i}{gm}$$

where *i* is the applied potential, *g* is the discharge part gradient, and *m* is the active material mass. Obviously, the *C_s_* value at the first cycle was found to be 36 F/g and followed by a dropped to 19.5 F/g as the cycle number increased to the 50th cycle. The decrease in the *C_s_* value could be due to not having very good contact between the electrolyte and electrodes [102,103,104]. In this work, the average value of specific capacitance (*C_s_*) was found to be ~19 F/g. The obtained *C_s_* value in this study is of great interest in comparison with those measured for other proton-based EDLC works. Our previous studies for CS:MC:NH_4_I [105], CS:Dextran:NH_4_F [106], and methylcellulose (MC):CS (CS):NH_4_I:glycerol systems displayed *C_s_* values of 1.76, 12, and 10 F/g, respectively [21]. Shuhaimi et al. [15] reported the *C_s_* value of 1.67 F/g for the system of MC-NH_4_NO_3_. It is obvious from Figure 13 that the Cs drops continuously with increasing the number of cycles. Anion ions from the NH_4_I salt possess a bigger radius, and it may begin blocking some of those ions in the electrode pores throughout its charge-discharge process. In its next charge-discharge step, these blocked ions will create a repulsive force to the same ions. Consequently, with the number of cycles, it could reduce the cyclic stability of the system [107].

The variation of the equivalence series resistance (ESR) of the fabricated EDLC versus cycle number is shown in Figure 13b. The ESR value of the EDLC was obtained via the equation below:

(17) $$ESR=\frac{V_{d}}{i}$$

where *V_d_* denotes the drop potential before the start of the discharging process, and *i* stands for the applied current. It is noteworthy that the value of the ESR acquired at the first cycle was found to be 177 Ω. It is also noticeable that the ESR value increases with the increment of cycle number, and attained the highest value of 300 Ω at the 350th cycle number, resulting from the maximum value of *V_d_*. The presence of internal resistance (*R_es_*) in the EDLC, which is also known as equivalence series resistance (ESR), can be related to the current collector, the charge-discharge procedure of electrolytes, and the distance between the polymer electrolyte and electrodes [3,108]. A low ESR value is promising for the applications of the EDLC. Besides, it can verify a strong interaction between electrolytes and electrodes through a low ESR value. The same trend in the ESR was recorded for the system of CS:MC:NH_4_I [105]. The system of CS:Starch:NH_4_I showed a very high ESR value compared to the current work [109]. Our previous study for the glycerolized PVA:NH_4_SCN:Cd(II)-complex showed the average ESR value of 51 Ω [2].

The energy density (*E_d_*) and power density (*P_d_*) are two crucial parameters that should be characterized to approve the application of the EDLC device. Figure 13c,d provide the *E_d_* and *P_d_* densities throughout 400 cycles for the highest conducting sample of the glycerolized CS:NH_4_I:Zn(II)-complex system. The *E_d_* and *P_d_* have been calculated using the equations in References [110,111].

The *E_d_* value in the first cycle was found to be 4.1 Wh/kg, while it dropped to 2.1 Wh/kg at the 50th cycle. Beyond the 50th cycle, the *E_d_* values are almost constant until 400th cycle, with an average of ~2 Wh/kg (see Figure 13c). At this point, ions subject the same energy barrier as it transports from the polymer electrolyte to the surface of the electrodes [112,113,114]. The ion accumulation at the electrode/electrolyte interfaces has a significant effect on the amount of energy storage in EDLC. Hamsan et al. [110] have stated an *E_d_* value of 2.25 Wh/kg using the potato starch (PS)-methylcellulose(MC)-NH_4_NO_3_-glycerol system. Our previous work for the CS:MC:NH_4_I:glycerol system showed an *E_d_* value of 0.77 Wh/kg, which is much lower than that reported in this study [21]. From Figure 13d, it was observed that the *P_d_* value at the first cycle is about 480 W/kg, while it decreased to 320 W/kg when it reached the 50th cycle as a result of an increase in the ESR and *V_d_*. Afterward, it was stable up to the 400th cycle, with an average value of ~300 W/kg. The *P_d_* value at the first cycle for the system of PVA-CS-NH_4_SCN-glycerol [16] was 399 W/kg, which is comparable with the result in this study.

## 4. Conclusions

The glycerolized CS:NH_4_I:Zn(II)-complex-based polymer composite electrolytes (PCEs) were prepared via the technique of cast solution. Polymer electrolytes are scientifically imperative in the fabrication of electrochemical SCs, fuel cells, batteries, and dye-sensitized solar cells. The ionic conductivity and dielectric properties were observed to be improved with increasing glycerol concentration. The sample incorporated with 30 wt.% of glycerol exhibited the highest dielectric constant with the maximum ionic conductivity of 1.17 × 10^−4^ S/cm. It was shown that when the glycerol amount increased, the diffusion coefficient (*D*), mobility (*μ*), and number density (*n*) of cations and anions fractions gradually increased. The Zn(II)-complex improved the EDLC performance because of the enhancement of the amorphous nature, whereas the high ionic conductivity is associated to the amorphous structure. It was indicated by the FESEM method that at the highest glycerol amount, the film has a smooth and homogenous surface morphology. It was indicated by the FTIR spectroscopy that there was an interaction of NH_4_I, Zn(II)-complex, and glycerol with the CS polymer by modifying the FTIR bands. Transference number measurements (TNM) confirmed that the dominant charge carriers were cations and anions fractions. The value of the cation and anion transference number (*t_ion_*) fraction was found to be 0.98. The electron transference number (*t_e_*) for the highest conducting electrolyte was identified to be 0.02. The measures of the LSV displayed that the CSNZG3 sample was decomposed at 1.37 V, suggesting the electrolyte suitability for the EDLC application. The CV curves showed no redox reaction peaks throughout its entire potential range. To evaluate the characteristics of the EDLCs composed of the glycerolized CS-based PCEs, cyclic voltammetry (CV) and the galvanostatic charge-discharge (GCD) curve were applied over 400 cycles. The fabricated EDLC showed that the initial value of specific capacitance (*C_s_*), equivalence series resistance (ESR), energy density (*E_d_*), and power density (*P_d_*) were found to be 36 F/g, 177 Ω, 4.1 Wh/kg, and 480 W/kg, respectively.

## Figures and Tables

**Table 1:** The composition and designation of glycerolized CS:NH_4_I: Zn(II)-complex systems.

Sample Designation	Wt. (g) CS	Wt. % NH_4_I	Zn (II)-Complex (mL)	Wt.% Glycerol
CSNZG0	1	40	10	0
CSNZG1	1	40	10	10
CSNZG2	1	40	10	20
CSNZG3	1	40	10	30

**Table 2:** DC ionic conductivity (*σ_dc_*) of the un-plasticized and plasticized CS:NH_4_I:Zn(II)-complex system at room temperature.

Sample Code	Ionic Conductivity (*σ_dc_*) (S. cm^−1^)
CSNZG0	1.56 × 10^−6^
CSNZG1	3.68 × 10^−6^
CSNZG2	1.01 × 10^−4^
CSNZG3	1.17 × 10^−4^

**Table 3:** The EEC fitting parameters for the plasticized systems at room temperature.

Sample	*K*_1_ (*F*^−1^)	*K*_2_ (*F*^−1^)	*C*_1_ (*F*)	*C*_2_ (*F*)
CSNZG0	4.6 × 10^8^	3.5 × 10^6^	2.17 × 10^−9^	2.86 × 10^−7^
CSNZG1	4.5 × 10^8^	1.90 × 10^6^	2.22 × 10^−9^	5.26 × 10^−7^
CSNZG2	-	2.7 × 10^5^	-	3.70 × 10^−6^
CSNZG3	-	1.85 × 10^5^	-	5.41 × 10^−6^

**Table 4:** The *ω*, *D*, *µ*, and *n* values at room temperature.

Sample	*ω* (rad s^−1^)	*D* (cm^2^ s^−1^)	*µ* (cm^2^ V^−1^ s)	*n* (cm^−3^)
CSNZG0	3.39 × 10^5^	4.13 × 10^−8^	1.60 × 10^−6^	6.06 × 10^18^
CSNZG1	2.01 × 10^6^	7.73 × 10^−8^	3.01 × 10^−6^	7.63 × 10^18^
CSNZG2	6.28 × 10^4^	3.65 × 10^−7^	1.42 × 10^−5^	4.43 × 10^19^
CSNZG3	6.16 × 10^5^	3.89 × 10^−7^	1.52 × 10^−5^	4.81 × 10^19^

**Table 5:** Specific capacitance (*C_p_*) value of the CSNZG3 film at the various scan rates of 100, 50, 20, and 10 mV/s.

Scan Rate (mV/s)	Area	Mass (g)	V_2_ − V_1_	Specific Capacitance (F/g)
100	6.70 × 10^−3^	2.43 × 10^−3^	0.9	15.32
50	4.22 × 10^−3^	2.43 × 10^−3^	0.9	19.29
20	2.20 × 10^−3^	2.43 × 10^−3^	0.9	25.14
10	1.38 × 10^−3^	2.43 × 10^−3^	0.9	31.55
